# Clinical Characteristics, Visual Outcomes, and Prognostic Factors of Open Globe Injuries

**DOI:** 10.3390/medicina57111198

**Published:** 2021-11-03

**Authors:** Edita Puodžiuvienė, Gabrielė Valeišaitė, Reda Žemaitienė

**Affiliations:** Department of Ophthalmology, Lithuanian University of Health Sciences, A. Mickevičiaus Str. 9, 44307 Kaunas, Lithuania; gabrvala0614@kmu.lt (G.V.); reda.zemaitiene@lsmuni.lt (R.Ž.)

**Keywords:** open globe injury, eye trauma, visual impairment

## Abstract

*Background and Objectives*: Open globe injuries (OGI) remain an important cause of visual impairment and loss, impacting all ages. A better understanding of the factors influencing visual outcomes is important in an attempt to improve the results of the treatment of OGI patients. The author aimed to contribute to this knowledge with the analysis of clinical characteristics, prognostic factors, and visual outcomes of their cohort of OGI patients. *Materials and Methods:* A retrospective medical record review was performed for 160 patients (161 eyes) who sustained an open globe injury between January 2015 and December 2017 and presented to the Hospital of Lithuanian University of Health Sciences. Data analyzed included age, sex, type, cause, place of OGI, initial visual acuity (VA), final best-corrected visual acuity (BCVA), and tissue involvement. Open globe injuries were classified using the Birmingham Eye Trauma Terminology (BETT) and Ocular Trauma Classification System (OTCS). Univariate analysis was conducted to evaluate the prognostic factors. *Results:* The mean age of the patients was 41.9 years. The male-to-female ratio was found to be 8.4:1. The home was the leading place of eye injury (59.6%), followed by an outdoor environment (14.3%) and workplace (11.8%). Penetrating injury accounted for 43.5%, followed by intraocular foreign body injury (39.1%) and globe rupture (13%). Overall, 19.5% of patients regained a good final vision of ≥0.5, but for 48.1% of them, eye trauma resulted in severe visual impairment (BCVA ≤ 0.02). In the univariate analysis, a bad visual outcome of less than 0.02 was correlated with bad initial VA, iris dialysis, hypotony, vitreous hemorrhage, and vitreous prolapse at presentation. Phthisis bulbi was correlated with eyelid laceration, iris prolapse, iris dialysis, hyphema, vitreous prolapse, vitreous hemorrhage, and choroidal rupture at initial examination. *Conclusions*: Open globe injury remains an important preventable cause of ocular morbidity. This study provides data indicating that open globe injuries are a significant cause of visual impairment in our research group.

## 1. Introduction

Open globe injury remains a major cause of permanent visual impairment and blindness in the world [1]. Despite advances in ophthalmic surgery and equipment, loss of vision may be unfavorable in a significant number of cases [1]. Open globe injury, defined as a full-thickness injury of the eyewall, presents severe damage to the eye and often results in poor outcomes [2]. The accumulation of knowledge about the pathophysiology of eye injuries and their prognostic factors as well as advances in diagnostic and therapeutic methods have greatly improved the success rates for managing open globe injuries [3]. A better understanding of these prognostic factors may help to provide our patients with better and more realistic expectations of their final visual acuity [3]. Many studies have been conducted to evaluate the factors associated with poor prognosis in patients with open globe injuries [1,3,4,5,6,7,8,9]. Several prognostic factors such as initial visual acuity, the involvement of ocular tissue, and both proper diagnosis and appropriate treatment may help to achieve a useful vision [1,3,4,5,6,7,8,9].

The current study aimed to evaluate the clinical characteristics of open globe injury patients presented to the Hospital of Lithuanian University of Health Sciences, the leading center for ophthalmic care in the country, and to identify the prognostic factors influencing the visual and structural outcomes of the treated patients.

## 2. Materials and Methods

This retrospective study reviewed the medical records of 160 patients who sustained an open globe injury (OGI) and were admitted to the Department of Ophthalmology of the Hospital of Lithuanian University of Health Sciences, from 1 January 2015 to 31 December 2017. The follow-up of patients occurred in the outpatient department of the same hospital.

Ethics statement. This retrospective study adhered to the tenets of the Declaration of Helsinki and received approval from the Regional Committee of Bioethics (No. BEC-MF-73; date of approval: 15 November 2017).

Clinical characteristics. Demographic features included age, gender, cause and place of injury, and date of injury were evaluated based on medical records. Initial VA, type of OGI, and initial diagnosis were recorded. Management (type and number of surgeries, time intervals from injury to admission, and from injury to surgery) and clinical data obtained at the end of follow-up periods such as final best-corrected visual acuity (BCVA) and final diagnosis were noted.

The study population was divided into three age groups: <18 years (children), 18–59 years (people of working age), and ≥60 years (people of retirement age). 

The classification of eye injuries was based on the Birmingham Eye Trauma Terminology (BETT) [10] and the Ocular Trauma Classification System (OTCS) [11] and categorized injuries by the following parameters: (1) type of OGI: globe rupture, penetrating injury, intraocular foreign body (IOFB) injury, perforating injury; (2) zone (wound location): zone 1—the cornea and limbus, zone 2—the anterior 5 mm of the sclera, zone 3—full-thickness scleral defects >5 mm posterior to the limbus; (3) grade (VA measurement on a Snellen acuity chart at the initial examination), as follows: grade 1 (≥0.5), grade 2 (0.2–0.4), grade 3 (0.03–0.1), grade 4 (light perception, LP-0.02), and grade 5 (no light perception, NLP). The final BCVA was classified according to the same method. Poor visual outcome was defined as final BCVA less than 0.02 (grade 4 and grade 5). 

Statistical analysis. The Statistical Package for Social Science (SPSS 22.0) was used for statistical analysis. Statistical analysis of all quantitative data including descriptive statistics, parametric, and nonparametric comparisons were performed for all variables. Chi-square independence (Fischer’s exact or Monte Carlo) test was performed to test differences in the proportions of categorical variables between two or more groups, and the Wilcoxon nonparametric test was used for dependent variables. The probability of an event given a certain risk factor was calculated using logistic regression analysis including the odds ratio (OR) and its confidence interval (95% CI). A *p* < 0.05 value was considered statistically significant.

## 3. Results

**Figure 1:**
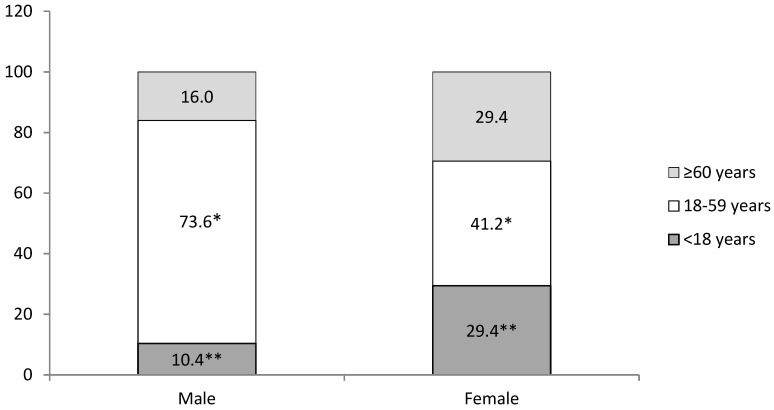
Distribution by age and gender (%). χ^2^ = 8.275, df = 2, *p* = 0.016; *, ** *p* < 0.05.

A total number of 160 patients (161 eyes), diagnosed with OGI, were admitted to the hospital during the study period. The age of patients at the time of injury ranged from three to 82 years with a mean of 41.9 ± 1.5 (M ± SE) years (42.2 ± 1.5 years in males and 39.1 ± 6.1 years in females). Male patients constituted 89.4% of the cases (*n* = 144), making the male-to-female ratio 8.4:1. Bilateral OGI was noted in one patient. Among the unilateral injuries, no significant difference was observed between the affected eyes (right eye 50.9% vs. left eye 49.1%, *p* > 0.05). The study population was divided into three age groups: <18 years (12.4%, *n* = 20), 18–59 years (70.2%, *n* = 113), and ≥60 years (17.4%, *n* = 28). A significant predominance of males in the age group of 18 to 59 years and females in the age group of <18 years was found. Figure 1 presents the details for age and gender distribution. 

Urban residents accounted for 47.2% of the total subjects, and residents from rural areas accounted for 52.8% (*p* > 0.05). Ten subjects (6.2%) admitted alcohol use before the injury.

Home was the leading place of eye injury (59.6%, *n* = 98), followed by outdoor environment (street/road, including traffic accidents) (14.3%, *n* = 23), workplace (11.8%, *n* = 19), recreation/sports (6.2%, *n* = 10), agriculture (2.5%, *n* = 4), school (0.6%, *n* = 1), and place unknown (5.0%, *n* = 8). No significant difference was obtained when analyzing place of injury and patient’s age.

The highest percentage of OGIs in all age groups was caused by sharp objects (61.5%, *n* = 99), followed by hammering on metal (14.9%, *n* = 24), blunt objects (11.8%, *n* = 19), lawn equipment (3.7%, *n* = 6), firework/explosion (3.1%, *n* = 5), fall (1.8%, *n* = 3), traffic accident (1.2%, *n* = 2), and cause unknown (1.8%, *n* = 3). No significant difference was also found between age groups concerning the cause of injury.

The most frequently reported sharp objects, in decreasing order of frequency, were metal fragments (30.4%, *n* = 49), sharp instruments (16.1%, *n* = 26), and glass/plastic fragments (8.7%, *n* = 14). Wooden sticks/firewood (8.1%, *n* = 13) and fist (8.1%, *n* = 13) were found to be the most common blunt objects. 

Regarding the type of injury, penetrating injury (43.5%, *n* = 73) accounted for the majority of OGIs, followed by IOFB injury (39.1%, *n* = 63), and globe rupture (13.0%, *n* = 21). Perforating injury (2.5%, *n* = 4) accounted for the remaining cases of OGI. 

Presenting VA was documented as NLP in all cases of perforating injuries. Three cases of these underwent primary wound closure and one eye was enucleated during the initial surgery. All patients with perforating injuries were lost for further follow-up. 

The distribution of IOFB by location and type was as follows: 39.7%—magnetic IOFB in the anterior segment, 7.9%—nonmagnetic IOFB in the anterior segment, 50.8%—magnetic IOFB in the posterior segment, and 1.6%—nonmagnetic IOFB in the posterior segment. 

There was a significant association between the type of OGI and age. The frequency of globe rupture was found to be significantly higher in the age group of 60 years and older (Table 1).

**Figure 2:**
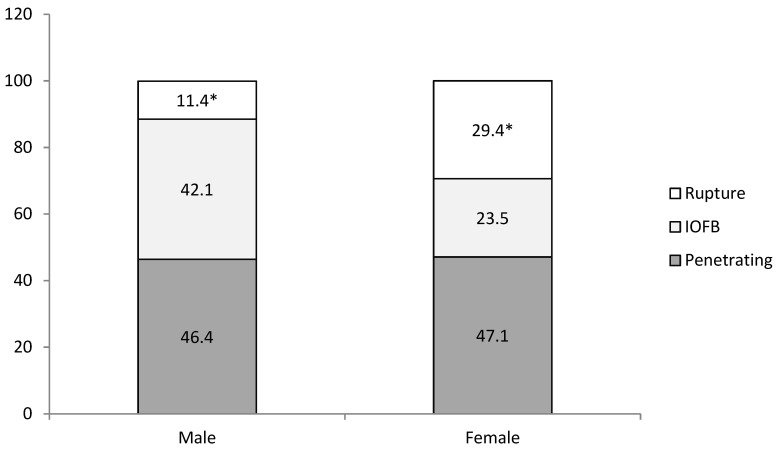
Distribution by gender and type of open globe injury (%). χ^2^ = 5.114, df = 2, *p* < 0.05; * *p* = 0.017; IOFB = intraocular foreign body.

Penetrating wound was found to be the most common type of OGI for both genders, but globe rupture significantly predominated among female patients (Figure 2). 

Through the cause of OGI, hammering on metal and injuries caused by lawn equipment were significantly associated with IOFB (38.1% and 7.9%, respectively), sharp objects—with penetrating injury and IOFB (93.2% and 46.0%, respectively), blunt objects, and fall—with globe rupture (90.5% and 9.5%, respectively) (χ^2^ = 216.131, df = 16, *p* < 0.001).

In terms of zone of injury, 75 (47.8%) eyes had zone 1 injury, 40 (25.5%) eyes had zone 2 injuries, and 42 (26.8%) eyes had zone 3 injuries. Zone 1 was significantly more frequently diagnosed with IOFB and penetrating injury versus rupture, and zone 3 with penetrating injury and rupture versus IOFB (Table 2).

The most common presentations of all OGIs were traumatic cataract (54.0%), hypotony (51.6%), vitreous hemorrhage (51.0%), hyphema (49.7%), uveal prolapse (49.7%), iris dialysis (34.2 %), iris laceration (39.8%), vitreous prolapse (28.0%), and retinal detachment (14.7%). Table 3 provides a comparison of initial diagnoses between the types of OGI.

Zone 3 injury was significantly related to such diagnoses determined on initial examination as eyelid laceration (*p* < 0.001), eyelid contusion (*p* = 0.001), uveal prolapse (*p* < 0.001), iris dialysis (*p* = 0.001), iris laceration (*p* < 0.001), lens dislocation (*p* = 0.049), hyphema (*p* < 0.001), hypotony (*p* < 0.001), vitreous prolapse (*p* < 0.001), choroidal hemorrhage (*p* = 0.001) versus zone 1 and zone 2, and retinal hemorrhage versus zone 1 (*p* = 0.045). Zone 2 injury was significantly associated with iris laceration (*p* < 0.001), iris dialysis (*p* = 0.001), lens dislocation (*p* = 0.049), and vitreous hemorrhage (*p* < 0.001) versus Zone 1. 

Grade of injury, depending on initial VA in our study, was as follows: grade 1—14.0% (*n* = 22), grade 2—12.1% (*n* = 19), grade 3—18.5% (*n* = 29), grade 4—47.1% (*n* = 74), and grade 5—8.3% (*n* = 13). A significant association between initial VA of grade 4 (LP-0.02) and globe rupture, grade 5 (NLP), and rupture/penetrating injury versus IOFB injury was found. The initial good VA of grade 1 (≥0.5) was significantly associated with IOFB injury (Table 2). 

In terms of zone of injury, the initial VA of grade 5 (NLP) was statistically significantly related to zone 3 injuries, and grade 2 (0.2–0.4) to zone 1 injury (Table 4).

Patients were admitted to the hospital on average 2.0 ± 0.2 days (range: from 1 to 30 days) after ocular injury: 60.9% (*n* = 98) during the first 24 h, 24.8% (*n* = 40) in 25–48 h, 5.0% (*n* = 8) in 49–72 h, and the remaining cases were admitted later.

All patients with OGI underwent surgery. Ninety-two eyes (57.1%) were operated on within the first 24 h and forty-nine eyes (30.4%) during 25–48 h after presentation. A primary procedure such as wound/rupture repair accounted for 54.7% of cases (88 eyes). Initial wound repair in combination with pars plana vitrectomy (PPV) was performed in 55 eyes (34.2%), initial PPV without wound repair in 16 eyes (9.9%), and lensectomy/cataract extraction in 31 eyes (19.3%). The IOFBs were removed during primary open globe repair in all 63 eyes with IOFB. Twenty-four eyes (14.9%) underwent secondary eye surgery. Types of secondary procedures included PPV (14 eyes), lensectomy/cataract extraction (10 eyes), and implantation of the intraocular lens (18 eyes). PPV, as a tertiary procedure, was performed in only one eye. Two eyes (1.2%) underwent primary enucleation. One eye was enucleated during secondary surgery. 

The patients were followed-up in the outpatient department with a mean period of 54.3 ± 4.5 days (from 14 to 162 days). The final results were found and evaluated in only 49.1% (*n* = 77) of all medical records, as the remaining eighty cases (50.9%) were lost to follow-up. The frequency of initial VA ≤ 0.02 (grade 4 and grade 5) was found to be similar for both groups (follow-up cases 59.0% versus lost cases 52.5%, *p* = 0.413). Among those seventy-seven follow-up cases, 19.5% (15 eyes) regained vision 0.5 and better. In six eyes (7.8%) final BCVA was 0.2–0.4, in 19 eyes (24.7%), 0.03–0.1, in 27 eyes (35.1%)—LP-0.02, and in 10 eyes (13.0%)—NLP. In summary, 48.1% of all follow-up cases were finished with poor visual outcomes. Nonparametric Wilcoxon Signed Rank Test did not reveal a significant difference between initial VA and final BCVA (Table 5).

Regarding the type of OGI, the final visual acuity of grade 5 (NLP) was significantly related to globe rupture versus IOFB injury. Grade 1 (≥0.5) was related to IOFB injury. In terms of zone of injury, zone 1 was significantly associated with the final BCVA of grade 2 (0.2–0.4), and zone 3 with the final BCVA of NLP. The distribution of final BCVA by zone and type of OGI is presented in Table 6. 

Univariate logistic regression analysis showed that an initial VA of 0.02 and worse was the strongest predictive factor of the poor visual outcome, with an odds ratio (OR) of 7.143 (95% confidence interval (CI), 2.519–20.257). Initial diagnoses such as iris dialysis (OR 2.783; 95% CI, 1.079–7.176), hypotony (OR 2.546; 95% CI, 1.006–6.443), vitreous hemorrhage (OR 3.125; 95% CI, 1.227–7.959), and vitreous prolapse (OR 3.069; 95% CI, 1.022–9.216) were also found to be significant predictive factors for the poor visual outcome of ≤0.02. Other factors such as injury zone, OGI type, retinal detachment, endophthalmitis, time of initial surgery, time from injury to presentation, and number of surgeries were not statistically significant in the univariate analysis.

The anatomical outcome was evaluated and documented at the last follow-up visit. The most common final diagnoses in decreasing order were corneal scars (68.8%), traumatic cataract (40.3%), scleral scars (37.7%), iris defects (27.3%), aphakia (24.7%), vitreous opacity (22.1%), vitreous hemorrhage (19.5%), retinal detachment (19.5%). Glaucoma (15.6%), hypotony (9.1%), phthisis bulbi (9.1%), lens dislocation (6.5%), and proliferative vitreoretinopathy (6.5%) accounted for lower number of cases.

Final diagnoses such as corneal scars were statistically significantly related with penetrating and IOFB injury versus rupture; scleral scar, vitreous hemorrhage, vitreous opacity, and lens dislocation with penetrating injury and rupture versus IOFB injury and choroidal rupture with globe rupture versus penetrating and IOFB injury (Table 7).

During the follow-up period, in seven (9.1%) eyes, phthisis bulbi, which was defined as an unfavorable anatomical outcome, was diagnosed. Four of those eyes suffered from penetrating injury, one eye had IOFB, and two eyes were ruptured. Eyelid laceration, iris prolapse, iris dialysis, hyphema, vitreous prolapse, vitreous hemorrhage, and choroidal rupture at initial presentation were statistically significant predictive factors for final phthisis bulbi by univariate logistic regression analysis (Table 8).

All factors found significant in the univariate logistic analysis were included in the multivariate logistic analysis to further evaluate their associations with the final VA and phthisis bulbi, but no statistically significant association was found because of the multicollinearity of those factors.

## 4. Discussion

This study aimed to evaluate the epidemiological, clinical characteristics, visual, and anatomical outcomes after severe open globe injuries in patients, presented to the Department of Ophthalmology of the Hospital of Lithuanian University of Health Sciences, the principal tertiary center for ocular injuries in the country, and to identify the possible prognostic factors, influencing the final visual and anatomical outcome.

The mean age of our population was 41.9 ± 1.5. This value is similar to those that have been reported in some other studies [5,6,12]. Many authors have reported the younger mean age of the patients [1,13,14,15,16], and this fact can be explained by different study designs or the specificity of the country.

We identified the predominance of the male gender for OGI (89.4%). This tendency has also been found in other epidemiological studies, with a male proportion varying between 66.0% and 96.7% [1,2,3,6,7,12,13,15,17,18,19,20,21,22]. This variation could be explained that men are at more risk of being exposed to dangerous situations in the workplace or during outdoor activities as well as during gender-based behavior [3]. Our study demonstrated that age was strongly associated with the incidence of OGI. The rate of OGI was found to be significantly higher in male patients in the age range between 18 and 59 years. These findings are consistent with those of other studies in which the peak age of male OGI ranged from 20 to 49 years (52.6%) [8], 21–50 years (55.03%) [6], and 41–60 years (40.9%) [16]. We also found that the risk of OGI was significantly higher for females younger than 18 years old. This finding could be explained by the fact that at a younger age, males and females are engaged in similar daily activities.

In our study, the home was the leading place of eye injury (59.6%), followed by an outdoor environment/street and workplace, which only accounted for 11.8% of all OGI. In line with our results, the previous literature has also reported that the home was the most frequently associated place of eye trauma [1,7,16,23]. Other studies have confirmed that the majority of open globe injuries are occupational, ranging from 22.0% to 50.0% of cases [1,2,3,4,6,13,19,20].

Injury by sharp objects is among the most common mechanisms of injury. Glass accounted for the majority of such injuries [4]. In our series, sharp objects such as metal fragments, sharp instruments, and broken glass, accounted for 61.5% of cases. These results are consistent with the data published by Rahman et al. [4]. In our series, hammering on metal was the second leading cause of injury and accounted for 14.9% of all causes of OGI, and this rate was higher than that reported by Makhrash et al. (8.3%) [7] and Rahman et al. (4%) [4]. Blunt objects accounted for 11.8% of all causes of OGI and were responsible for ninety percent of globe ruptures in our study. Other studies reported a blunt mechanism to be responsible for 28% [4] and 20% [7] of all OGI cases. Fall was found to be one of the main causes of the blunt mechanism of injury, especially in older female patients [3], significantly predominated in older age groups [24]. Our results could not prove this fact, but we found a significant risk for globe rupture in older women.

In our study, penetration (43.5%) was the most common type of injury, followed by IOFB (39.1%) and rupture (13%). Similarly, Batur et al. [13] and Bauza et al. [19] also found that penetration (61.5% and 61.2%, respectively) was the most common type of OGI, followed by IOFB, 16.1% and 20.8%, respectively). Rahman et al. [4] and Fujikawa et al. [3] reported that rupture (56% and 69.5%, respectively) was the most common type of injury, followed by penetration (32%) and IOFB (9%) [4] or IOFB (20.3%) and penetration (10.2%) [3]. Similar to the literature, in our series, perforating injuries were rare (2.5%). The reported rate of perforating injury varied from 0.7% to 26.7% [4,5,7,13,14].

An analysis of the zones of injury revealed that almost half of our patients had zone 1 injury (47.8%) and more than one-fourth of them had zone 3 injuries (26.8%). Similar data were presented by Wang et al. [16]. Numerous studies found similar rates for zone 1, which was the most common location of the injury, but in contrast, zone 2 was indicated as the second leading location of injury in these studies [1,5,7,8,13,14,19]. Furthermore, in our series, zone 3 injury was significantly related to initial diagnoses such as eyelid laceration, eyelid contusion, uveal prolapse, iris dialysis, iris laceration, lens dislocation, hyphema, hypotony, vitreous prolapse, choroidal hemorrhage, and initial VA of grade 5 at initial examination. The current investigation demonstrated that the zone of injury was also found to be associated with visual outcomes. Wounds involving zone 3 had significantly poorer presenting and final VA versus those involving zones 1 or 2. These results are supported by previous studies that have reported a significant association between the posterior extension of injury and a worse final VA [2,3,7,8,9,13]. Regarding the mechanism of injury, our study confirmed that globe rupture was associated with a lower rate of visual survival and functional success than a laceration. These results are consistent with the data published by Fujikawa et al. [3] and Feng et al. [21].

Analysis of OGI in our study showed that traumatic cataract (54.0%), hypotony (51.6%), vitreous hemorrhage (51.0%), hyphema (49.7%), uveal prolapse (49.7%), iris dialysis (34.2%), iris laceration (39.8%), vitreous prolapse (28.0%), and retinal detachment (14.7%) were common presentations of all OGIs, which is consistent with results from other studies [1,5,7,14,17,22]. According to our results, eyelid contusion, lens dislocation, vitreous hemorrhage, and retinal detachment were the initial diagnoses significantly associated with globe rupture in comparison with other types of OGI. Eyelid laceration, eyelid contusion, lens dislocation, hyphema, hypotony, vitreous prolapse, uveal prolapse, vitreous prolapse, vitreous hemorrhage, and choroidal hemorrhage were significantly associated with zone 3 injury compared to zone 1 and zone 3. These outcomes could be related to the fact that the association between globe rupture, zone 3 of injury, and the wide spectrum of intraocular tissue injury reflects the seriousness of the ocular damage, in agreement with Agrawal et al. [25].

In our study, 14.0% of all OGIs presented with an initial VA of grade 1 (≥0.5). Initial VA of ≤0.02 (grades 4 and 5) accounted for 55.3% of injuries at presentation. These findings were consistent with the studies performed by Meng et al. [6], Fu et al. [12], and Bauza et al. [19]. In contrast to these results, Pimolrat et al. reported that presenting VA less than 6/60 was determined in 92% of cases [26].

In our series, initial grade 1 was significantly related to IOFB injury. Grade 2 was significantly associated with penetrating injury, IOFB, and zone 1. Grade 4 (0.02-LP) showed a significant association with globe rupture. Grade 5 (NLP) was significantly related to rupture and penetrating injury versus IOFB and to zone 3 of injury.

Analysis of initial and final visual acuity was evaluated and compared in seventy-seven cases of follow-up patients. No statistically significant difference was found between the grades of injury at the initial and final examination. In our study, 19.5% of injured eyes regained a good vision of ≥0.5. Comparisons with other studies are complicated due to the differences in study design and the great variability in the nature and severity of eye injuries themselves. Other studies reported achieving ≥0.4 in 29% [18], ≥0.5 in 22.29% [6], 26.8% [13], and 38.5% [19] of patients.

In our study, 48.1% of patients reached a final BCVA of 0.02 or less, but here again, a great disparity of results was observed as Meng et al. reported 29.39% (HM/LP) and 5.1% (NLP) [6], Batur et al. 34.4% (≤0.02) [13], and Bauza et al. 12.4% (HM/LP) and 12.4% (NLP) [19].

Regarding the type of OGI, the final visual acuity of grade 5 (NLP) was significantly related to globe rupture. A good visual outcome of grade 1 (≥0.5) was significantly related to IOFB injury. In contrast, the analysis presented by Atic et al. showed a strong association between IOFB and poor visual outcomes [1]. Our results also demonstrated that the zone of injury was associated with visual outcomes. Wounds involving zone 3 had significantly bad visual outcomes of NLP versus those involving zones 1 or 2. These findings were supported by numerous investigations [1,3,8,13,19].

In univariate analysis, an initial visual acuity of ≤0.02 (grades 4 and 5) was significantly associated with the final poor visual outcome of ≤0.02. The univariate analysis also demonstrated that the presence of iris dialysis, hypotony, vitreous hemorrhage, and vitreous prolapse was significantly associated with final BCVA ≤0.02 (grades 4 and 5) in this study. However, multivariate analysis did not reveal differences in these predictive factors, conceivably because of their multicollinearity.

In agreement with our results, initial visual acuity ≤0.02 [3,4,6,7,8,9,19] and vitreous hemorrhage [3,7] were reported to be an important predictive factor of the poor visual outcome by other researchers. The findings of numerous studies indicated that globe rupture [3], presence of retinal detachment [3,7,8], dislocation of the crystalline lens [3], presence of RAVD [3,8], larger wound (>10 mm) [8], zone 3 injury [3,7,19], and aphakia [7] were the most significant predictors of final visual outcome, determined in univariate or multivariate analysis. Our study did not find a statistically significant association between these factors and visual prognosis. This could be explained by different study designs, the great variability in the nature and severity of the eye injuries themselves, or by the particularities of countries where the investigation occurred.

According to our results, diagnoses defined at the last follow-up visit such as corneal scars, glaucoma, traumatic cataract, vitreous opacities, PVR, and retinal detachment were mostly related to globe rupture and penetrating injury. A traumatic cataract is the most common vision limiting complication, and it can occur any time from day 1 to several years after OGI [1]. We found that there was no significant association between traumatic cataracts and poor visual outcomes. These results are supported by data published by Atic et al. [1], but, in contrast, Fujikawa et al. found a significant correlation between lenticular involvement and bad visual outcome [3]. Atic et al. reported that retinal detachment was found to be a predictor of poor outcome [3], but our results could not prove this finding.

In terms of the type of OGI, IOFB injury showed the best anatomic success in comparison with penetration and rupture in our study.

In the current investigation, seven eyes ended up with phthisis bulbi at the end of the follow-up period, two eyes underwent primary enucleation, and one eye was enucleated at a subsequent surgical procedure. Feng et al. reported 15 cases of NLP, enucleation, or phthisis [21], Souylu et al. found that in 17.7% of eyes, phthisis bulbi occurred during the follow-up period [18].

Phthisis bulbi and enucleation were defined to present an unfavorable anatomic and visual outcome, significantly associated with predictive factors such as rupture, zone 3, large scleral wound, ciliary body damage, severe intraocular hemorrhage, closed funnel retinal detachment and retinal prolapse, and choroidal hemorrhage [21]. Univariate logistic regression analysis showed that eyelid laceration, iris prolapse, iris dialysis, hyphema, vitreous prolapse, vitreous hemorrhage, and choroidal rupture at initial presentation were statistically significant predictive factors for final phthisis bulb, in our study. Other studies also concluded that vitreous hemorrhage was also a predictor of poor outcome.

Rahman et al. reported a higher rate of secondary enucleations (12%) after OGI. They found RAVD, the presence of lid laceration, a blunt mechanism of injury, and initial VA worse than 6/60 on presentation to be significant risk factors associated with eventual enucleation [4]. In our series, two eyes underwent primary and one eye secondary enucleation, and no significant associations between clinical factors and enucleation were found.

Limitations of our study need to be noted. First, it was conducted as a retrospective study design and contained a considerable number of unrecorded data. Second, the study was related to variable follow-up times, and in some of the cases, the follow-up duration was relatively short.

## 5. Conclusions

In conclusion, a patient with OGI should be carefully examined both at the time of admission and during the follow-up period. This study confirms that some clinical characteristics such as initial visual acuity, iris dialysis, hypotony, vitreous hemorrhage, and vitreous prolapse may have the potential to correctly predict final visual outcomes. In the future, a prospective study of OGI, aimed at a more detailed evaluation of prognostic factors and prediction of functional outcomes, could provide solid evidence for building better management strategies in cases of OGI.

## Figures and Tables

**Table 1:** Distribution by age and type of open globe injury (OGI).

Type of OGI	Age Group (Years); *n* (%)		
	<18	18–59	≥60
Penetrating	11(57.9)	53(47.7)	9(33.3)
IOFB	7(36.8)	46(41.4)	10(37.0)
Rupture	1(5.3) **	12(10.8) ***	8(29.6) **, ***

χ^2^ = 8.551, df = 4, *p* = 0.01; **^,^ ***p < 0.05; IOFB = intraocular foreign body, OGI = open globe injury.

**Table 2:** Types of open globe injuries classified by zone and grade.

Zone/Grade of Injury	Type of OGI; *n* (%)		
	Penetrating	IOFB	Rupture
Zone			
Zone1	30(41.1) *^,^ **	45(71.4) *^,^ ***	0(0) **^,^ ***
Zone 2	16(21.9)	15(23.8)	9(42.9)
Zone 3	27(37.0) *	3(4.8) *^,^ ***	12(57.1) ***
χ^2^ = 42.512, df = 4, *p* < 0.001; *^,^ **^,^ *** *p* < 0.05			
Grade (initial VA)			
1 (≥ 0.5)	4(5.5) *^,^ **	16(25.4) *^,^ ***	2(9.5) **^,^ ***
2 (0.2–0.4)	7(9.6) *^,^ **	12(19.0) *^,^ ***	0(0.0) **^,^ ***
3 (0.03–0.1)	15(20.5)	12(19.0)	2(9.5)
4 (LP-0.02)	38(52.1) **	23(36.5) ***	13(61.9) **^,^ ***
5 (NLP)	9(12.3) *	0(0.0) *^,^ ***	4(19.0) ***

IOFB = intraocular foreign body, LP = light perception, OGI = open globe injury, NLP = no light perception, Zone 1—cornea and limbus, Zone 2—limbus to 5 mm posterior into sclera, Zone 3—posterior to 5 mm from the limbus, VA = visual acuity. χ^2^ = 28.906, df = 8 *p* < 0.001, *^,^ **^,^ *** *p* < 0.05.

**Table 3:** Distribution of initial diagnoses by type of open globe injury.

Initial Diagnoses	Type of OGI; *n* (%)			
	Penetrating	IOFB	Rupture	χ^2^, df = 2, *p*
Eyelid: wound	32(43.8) *	8(12.7) *^,^ ***	10(47.6) ***	17.884; <0.001
Eyelid: contusion	35(47.9) *^,^ **	4(6.3) *^,^ ***	17(81.0) **^,^ ***	43.163; <0.001
Uveal prolapse	50(68.5) *	13(20.6) *^,^ ***	14(66.7) ***	34.005; <0.001
Iris: dialysis	36(49.3) *	6(9.5) *^,^ ***	12(57.1) ***	29.288; <0.001
Iris: laceration	45(61.6) *	5(7.9) *^,^ ***	12(57.1) ***	43.982; <0.001
Hyphema	45(61.6) *	18(28.6) *^,^ ***	16(76.2) ***	21.287; <0.001
Hypotony	53(72.6) *	12(19.0) *^,^ ***	16(76.2) ***	44.709; <0.001
Lens: cataract	41(56.2)	33(52.4)	11(52.4)	0.225; 0.894
Lens: dislocation	8(11.0) *^,^ **	1(1.6) *^,^ ***	9(42.9) **^,^ ***	26.462; <0.001
Vitreous: hemorrhage	37(50.7) **	27(42.9) **^,^ ***	16(76.2) **^,^ ***	7.007; 0.03
Vitreous: prolapse	41(43.8) *	3(4.8) *^,^ ***	10(47.6) ***	29.511; <0.001
Retina: laceration	6(8.2) **	11(17.5)	6(28.6) **	6.067; 0.048
Retina: detachment	6(8.2) **	9(14.3)	6(28.6) **	5.905; 0.016
Uveitis	17(23.3) **	15(23.8) ***	0(0.0) **^,^ ***	6.212; 0.045
Endophthalmitis	3(4.1)	2(3.2)	1(4.8)	0.139; 0.933

*^,^ **^,^ *** *p* < 0.05; IOFB = intraocular foreign body, OGI = open globe injury.

**Table 4:** Distribution by zone and grade of open globe injury.

Grade of Injury (Initial VA)	Zone of Injury; *n* (%)		
	Zone 1	Zone 2	Zone 3
1 (≥0.5)	12(15.4)	7(17.5)	3(7.0)
2 (0.2–0.4)	13(16.7) **	5(12.5)	1(2.3) **
3 (0.03–0.1)	19(24.4)	6(15.0)	5(11.6)
4 (LP-0.02)	33(42.3)	19(47.5)	23(53.5)
5 (NLP)	1(1.3) **	3(7.5) ***	11(25.6) **^,^ ***

χ^2^ = 28.157, df = 8 *p* < 0.001; VA = visual acuity, LP = light perception, NLP = no light perception, Zone 1—cornea and limbus, Zone 2—limbus to 5 mm posterior into sclera, Zone 3—posterior to 5 mm from the limbus. ** Zone 1 vs. Zone 3, *** Zone 2 vs. Zone 3.

**Table 5:** Distribution of initial and final visual acuity.

Grade of Injury	Initial VA *n* (%)	Final BCVA
1 (≥0.5)	10(13.0)	15(19.5)
2 (0.2–0.4)	10(13.0)	6(7.8)
3 (0.03–0.1)	12(15.6)	19(24.7)
4 (LP-0.02)	41(53.2)	27(35.1)
5 (NLP)	4(5.2)	10(13.0)

z = 1.109, *p* = 0.267 (non-parametric Wilcoxon signed-rank test); BCVA = best-corrected visual acuity, LP = light perception, NLP = no light perception, VA = visual acuity.

**Table 6:** Distribution of clinical factors by final best-corrected visual acuity.

Type/Zone of Injury	Grade (Final BCVA)				
	1 (≥0.5)	2 (0.2–0.4)	3 (0.03–0.1)	4 (LP-0.02)	5 (NLP)
Type					
Penetrating	1(3.1) *	5(15.6)	5(15.6)	19(59.4)	2(6.3)
IOFB	8(25.8) *	5(16.1)	5(16.1)	13(41.9)	0(0.0) **
Rupture	1(7.1)	0(0.0)	2(14.3)	9(64.3)	2(14.3) **
χ^2^ = 14.131, df = 8 *p* = 0.047; *^,^ ** *p* < 0.05; * penetrating vs. IOFB					
Zone					
Zone 1	6(15.4)	9(23.1) *	7(17.9)	17(43.6)	0(0.0) **
Zone 2	3(18.8)	0(0.0) *	2(12.5)	10(62.5)	1(6.3)
Zone 3	1(4.5)	1(4.5)	3(13.6)	14(63.6)	3(13.6) **
χ^2^ = 14.884, df = 8 *p* = 0.004; *^,^ ** *p* < 0.05; * zone 1 vs. zone 2, ** zone 1 vs. zone 3					

BCVA = best-corrected visual acuity, IOFB = intraocular foreign body, LP = light perception. NLP = no light perception, Zone 1—cornea and limbus, Zone 2—limbus to 5 mm posterior into sclera, Zone 3—posterior to 5 mm from the limbus.

**Table 7:** Final diagnosis according to the type of open globe injury.

Final Diagnosis	Type of OGI; *n* (%)			χ^2^; df = 2; *p*	
	Penetrating, *n* = 32	IOFB, *n* = 31	Rupture, *n* = 14		
Corneal scars	26(81.3) **	22(71.0) ***	5(35.7) **^,^ ***	9.523	0.009
Scleral scars	14(43.8) *	5(16.1) *^,^ ***	10(71.4) ***	13.426	0.001
Glaucoma	4(12.5)	3(9.7) ***	5(35.7) ***	5.366	0.049
Hypotony	3(9.4)	3(9.7)	1(7.1)	0.08	0.961
Phthisis	4(12.5)	1(3.3)	2(14.3)	2.197	0.333
Traumatic cataract	10(31.3)	13(41.9)	9(64.3)	4.379	0.112
Dislocated lens	2(6.3) *^,^ **	0(0.0) *^,^ ***	3(21.4) **^,^ ***	7.299	0.026
Aphakia	6(18.8)	10(32.3)	3(21.4)	1.643	0.44
Vitreous hemorrhage	7(21.9) *	1(3.2) *^,^ ***	7(50.0) ***	13.652	0.001
Vitreous opacity	11(34.4) *	0(0.0) *^,^ ***	6(42.9) ***	15.11	0.001
Retinal detachment	7(21.9)	4(12.9)	4(28.6)	1.71	0.425
PVR	1(13.1)	2(6.5)	2(14.3)	1.998	0.368
Choroidal rupture	0(0.0) **	0(0.0) ***	2(14.3) **^,^ ***	9.24	0.01

*^,^ **^,^ *** *p* < 0.05; IOFB = intraocular foreign body, OGI = open globe injury.

**Table 8:** Relationship between clinical factors and phthisis bulbi.

Clinical Factors for Phtisis Bulbi	No; *n* (%) *n* = 70/24 *	Yes; *n* (%) *n* = 7	*p*-Value	OR (%) [95% CI]
Eyelid: laceration	27.1/25.0 *	85.7	0.002/0.004 *	16.105 [1.818–142.695]
Iris: prolapse	45.7/50 *	100	0.06/0.017 *	-
Iris: dialysis	34.3/41.7 *	85.7	0.08/0.04 *	11.5 [1.308–101.101]
Hyphema	47.1/50.0 *	100	0.08/0.017 *	-
Vitreous: hemorrhage	48.6/54.2 *	100	0.09/0.026 *	-
Vitreous: prolapse	20.0/33.3 *	71.4	0.03/0.072 *	10.0 [1.753–57.046]
Choroid: rupture	2.9/0.0 *	28.6	0.03/0.07 *	13.6 [1.568–117.945]

OR—odds ratio, CI—confidence interval, * Random sample of 24 cases to evaluate the significance.

## Data Availability

The data used to support the findings of this study are available from the corresponding author upon request.

## Abbreviations

BCVA: best-corrected visual acuity; BETT: Birmingham Eye Trauma Terminology; CI: confidence interval; LP: light perception; NLP: no light perception; OGI: open globe injury; OR: odds ratio; OTCS: Ocular Trauma Classification System; PPV: pars plana vitrectomy; PVR: proliferative vitreoretinopathy; VA: visual acuity.
